# Leveraging Self-Attention Mechanism for Attitude Estimation in Smartphones

**DOI:** 10.3390/s22229011

**Published:** 2022-11-21

**Authors:** James Brotchie, Wei Shao, Wenchao Li, Allison Kealy

**Affiliations:** 1 School of Science, RMIT, Melbourne, VIC 3000, Australia; 2 School of Electrical and Computer Engineering, UC Davis, Davis, CA 95616, USA; 3 Victorian Department of Environment, Land, Water and Planning, Melbourne, VIC 3000, Australia

**Keywords:** attitude estimation, deep learning, inertial measurement unit, self-attention, smartphone

## Abstract

Inertial attitude estimation is a crucial component of many modern systems and applications. Attitude estimation from commercial-grade inertial sensors has been the subject of an abundance of research in recent years due to the proliferation of Inertial Measurement Units (IMUs) in mobile devices, such as the smartphone. Traditional methodologies involve probabilistic, iterative-state estimation; however, these approaches do not generalise well over changing motion dynamics and environmental conditions, as they require context-specific parameter tuning. In this work, we explore novel methods for attitude estimation from low-cost inertial sensors using a self-attention-based neural network, the Attformer. This paper proposes to part ways from the traditional cycle of continuous integration algorithms, and formulate it as an optimisation problem. This approach separates itself by leveraging attention operations to learn the complex patterns and dynamics associated with inertial data, allowing for the linear complexity in the dimension of the feature vector to account for these patterns. Additionally, we look at combining traditional state-of-the-art approaches with our self-attention method. These models were evaluated on entirely unseen sequences, over a range of different activities, users and devices, and compared with a recent alternate deep learning approach, the unscented Kalman filter and the iOS CoreMotion API. The inbuilt iOS had a mean angular distance from the true attitude of $117.31^{\circ }$, the GRU $21.90^{\circ }$, the UKF $16.38^{\circ }$, the Attformer $16.28^{\circ }$ and, finally, the UKF–Attformer had mean angular distance of $10.86^{\circ }$. We show that this plug-and-play solution outperforms previous approaches and generalises well across different users, devices and activities.

## 1. Introduction

Advancements in micro-electromechanical systems have eventuated in miniaturised Inertial Measurement Units (IMUs) that have increasingly low cost and power requirements. This has facilitated their ubiquity in modern electronics, such as smartphones. As such, the processing and evaluation of IMU signals as a means of motion tracking is a crucial component for many applications; most notably in inertial navigation [1], satellite control [2], space junk estimation [3], augmented reality and human body motions [4]. In order to estimate the motion of a rigid body from raw IMU measurements, one needs to first determine the attitude of said body with respect to some inertial reference frame—most commonly the Earth’s local frame.

In this paper, we focus on approaches that use triaxial measurements from three inertial sensors, commonly found in smartphones, and leverage the continuously provided information to estimate the attitude of the rigid sensor body with respect to the Earth’s local frame. These IMUs typically consist of a triaxis accelerometer, gyroscope and magnetometer. Directional vector observations can be taken from accelerometers and magnetometers, whereas gyroscopes provide angular velocities. Integration of the angular velocity measurements unfortunately leads to increasingly large errors in attitude estimation due to the sensor bias. An in-depth look into a wide range of IMUs and their deficiencies can be found in [5]. As the integration of gyroscope measurements yields poor estimations, traditional estimation techniques use accelerometer and magnetometer measurements to update error calculations and compensate for the drift. The generalised problem for attitude estimation from IMUs is in the combination of these sensors to provide an optimal solution in the form of an optimal-state estimator.

Most of the complexity in attitude estimation stems from its nonlinearity, and therefor its estimation solution must account for the nonlinear dynamics in the system. Early applications relied on the extended Kalman filter (EKF) to linearise the dynamic system about the current best-state estimate; however, this process can yield poor performance particularly in highly dynamic situations due to divergence and constant reinitialisation [6]. These difficulties led to the development of alternative filters, several of which retain the basic structure of the EKF; most notably the Unscented Kalman Filter (UKF) which, at the time of writing, is the industry standard. A survey of nonlinear attitude estimators is found in [7]. Despite all the iterative improvement to Kalman-filter-based estimators over the years, they are still dependent upon system model assumptions, and a deviation from defined assumptions may lead to divergence or failure of the system [6,7]. The shared reliance on a set of parameters that need to be predetermined or situationally adjusted in order to achieve satisfactory results have such a profound influence over performance that entire bodies of work have been built around calculating these values optimally [8]. Initial formulations had these tuned manually by trial and error methods. This is primarily due to the hardware complexity of consumer-grade IMUs, as it becomes almost infeasible for researchers and engineers to formulate the exact mathematical equations to describe the sensor noise and intrinsic models. Generalisability across the array of variables in applications that rely on attitude estimation is of great importance. Therefore, using data-driven methods instead of the model-based ones in this domain could improve our solutions.

Artificial Intelligence (AI) has demonstrated the advantages in utilising computing resources and data over traditional human understanding, predominately in computer vision [9] and natural language processing (NLP) [10]. The ability to employ continuous activation functions and their inherent understanding of time allows them to accurately model system complexities and interpolate in high-dimensional spaces [11]. Recent work employing self-attention-based deep learning (DL) networks in time-series forecasting [12], image recognition/production [13], text summarisation [14], speech recognition [15] and music generation [16] has shown state-of-the-art performance in terms of robustness and accuracy. Self-attention allows for the network inputs to interact with one another and be scored based on their correlation with their importance to the final estimate. This formulation has been extensively researched, however, little work has been conducted using the raw sequential measurements from low-cost noisy inertial sensors to learn deep attitude estimation. The success seen in other sequence-to-sequence learning tasks suggests that implementation of self-attention-based DL methodologies could obviate the need for precise sensor noise models and provide a more robust estimation.

The main contribution of this paper is to present a novel methodology for attitude estimation from low-cost inertial sensors. We propose leveraging self-attention mechanisms to learn the noise and bias characteristics of inertial sensors over different activities, users and devices. Providing a generalisable, end-to-end and out-of-the-box solution for attitude estimation in smartphones and low-cost IMUs.

The rest of the paper is arranged as follows: the related literature is given in Section 2. We formulate the problem and present a detailed description of our methodology in Section 3. Our results and analysis are given in Section 6. Finally, we draw some conclusions and delineate potential future work in Section 7.

## 2. Related Work

The recent literature has shown that DL networks have been utilised to regress attitudes from IMU measurements, as well as augment conventional techniques. In [17], Brossard et al. use a convolutional neural network (CNN) to compensate for the measurement error in the gyroscope. The authors of [18,19] use some formulation of an artificial neural network to compensate for residual errors in conventional attitude estimation algorithms and, in [20], DL is used to estimate the noise parameters used in said algorithms. A number of end-to-end solutions have been proposed using Recurrent Neural Networks (RNNs). The authors of [21,22] use an RNN based on Long Short-Term Memory (LSTM) to propagate the state. In [23], an LSTM is used in tandem with an EKF to stabilise the network output. Finally, Weber et al. propose RIANN (Robust IMU-based Attitude Neural Network) in [24], using a variation on LSTM, the Gated Recurrent Unit (GRU).

The methods that have proposed end-to-end attitude estimation solutions have primarily focused on RNNs using data from only the gyroscope and accelerometer measurements, or have been supplemented by conventional techniques. These canonical DL approaches only capture short-term directional information and are unable to retain information and dependencies over long sequences. In this work, we look at using a self-attention-based attitude estimation model based on encoder–decoder networks [25], as they are able to process long sequences whilst retaining important contextual information. To our knowledge, our approach is the only end-to-end attitude estimation solution that leverages all available inertial information.

## 3. Problem Formulation

This paper considers the problem of attitude estimation from low-cost IMUs, commonly found in smartphones. It is implicit that these systems are characterised by high noise levels and time-varying additive biases. The available measurements from a typical smartphone IMU are from three-axis rate gyros, three-axis accelerometers and three-axis magnetometers. The reference frame of the IMU is termed the body frame (B), which is rotated with respect to some fixed intertial frame (I), e.g., an inertial reference frame. The rotation R=RBI denotes the relative attitude of *B* with respect to *I*.

### IMU Model

The rate gyro measures the angular velocity of *B* relative to *I*, expressed in the bodies’ frame of reference, *B*. The error model is commonly given by [26]

(1) $$\begin{array}{cc} g & =\tilde{g}+\beta +\eta_{v}\in R^{3}\\ \dot{\beta } & =\eta_{u} \end{array}$$

where g denotes the measured angular rate, β is the gyro drift rate and $\eta_{v}$ and $\eta_{u}$ denote the independent zero-mean Gaussian white noise processes

(2) $$\begin{array}{cc} \; & E\left\{\eta_{v}\left(t\right)\eta_{v}^{T}\left(\tau \right)\right\}=\sigma_{v}^{2}\delta \left(t-\tau \right)I_{3\times 3}\\ \; & E\left\{\eta_{u}\left(t\right)\eta_{u}^{T}\left(\tau \right)\right\}=\sigma_{u}^{2}\delta \left(t-\tau \right)I_{3\times 3} \end{array}$$

where E{·} denotes expectation and δ(·) is the Dirac delta function, where $\sigma_{v}^{2}$ and $\sigma_{u}^{2}$ are scalars that satisfy $R=E\left[n_{i+1}^{2}\right]=\sigma_{n}^{2}$.

The accelerometer measures the linear acceleration of *B* relative to *I*, expressed in *B*. As with the rate gyro, the output from a MEMS component accelerometer has added noise and bias,

(3) $$a=R^{T}\left(\dot{v}-G_{0}\right)+\beta_{a}+\eta_{a}$$

where $\beta_{a}$ is the bias term, $\eta_{a}$ denotes additive measurement noise and $G_{0}$ represents the gravitational acceleration field.

Finally, the magnetometer provides measurements of the magnetic field

(4) m=RTmA+βm+ηm

where mA denotes the Earth’s magnetic field, $\beta_{m}$ is a body-fixed representation of the local magnetic disturbance and $\eta_{m}$ is the measurement noise.

If we consider the accelerometer and magnetometer measurement vectors, we can construct an instantaneous algebraic measurement, $R_{y}$, of the rotation RBI [27]

(5) $$\begin{array}{cc} R_{y} & =\arg\min_{R\in \mathrm{SO}(3)}\left(\lambda_{1}{∥e_{3}-R\frac{a}{∥a∥}∥}^{2}+\right.\\ \; & \;\left.\lambda_{2}{∥m^{*}-R\frac{m}{∥m∥}∥}^{2}\right) \end{array}$$

(6) ≈RBI

where ∥·∥ is the 2-norm, $m^{*}$ is the localised inertial direction of the magnetic field, $e_{3}$ is the normalised gravity vector and $\lambda_{1}$ and $\lambda_{2}$ are weights chosen based on sensor output confidence. SO(3) is the special orthogonal group defined by $\left\{R\in R^{3}|RR^{T}=I_{3},\det\left(R\right)=\pm 1\right\}$.

For the algebraic measurement given in Equation (5), two degrees of freedom in the rotation are resolved using the accelerometer readings (Equation (3)), and the final degree of freedom is resolved by the magnetometer (Equation (5)). This results in the error properties of the reconstructed attitude, $R_{y}$, being difficult to characterise, and if at any point either of the readings are unavailable, then the algebraic attitude measurement becomes impossible to resolve [27]. To overcome this, many statistical models have been introduced where the state estimate is formed by propagating the IMU readings through measurement and kinematic models [28]; we formulate the Unscented Kalman filter used in this work in Section 4.3.2. However, the reality for these traditional estimation approaches is that the hand curation and rigidity severely limit their performance and generalisability. Additionally, measurement imperfections, inaccurate system modelling, unrealistic requirements and complex dynamics impair the accuracy and reliability. An AI approach requires no prior information.

## 4. Proposed Solutions

Modern learning methods allow machine intelligence systems to learn from past experience and actively exploit new information without having to explicitly specify the complex mathematical and physical constructs. This has potential for the discovery of novel computational solutions to the optimisation problem. This work parts from the commonly used eschew recurrence in neural networks, used by related work (Section 2), and instead relies entirely on a self-attention mechanism to draw global dependencies between inputs and outputs. A disadvantage in this approach over traditional algorithms is the lack of prior estimate in the determination of the current, which could lead to large outlier estimates for periods where large spikes of noise are found in the measurements. To avoid this, we also evaluated the combination of UKF priors in the self-attention NN learning process. In addition to self-attention-based NNs, this section details the mathematical and baseline estimation solutions.

### 4.1. Parameterisation

It can be shown that all attitude representations in $R^{3}$ suffer from nonuniqueness, discontinuity in the representation space and singularities (commonly referred to as gimbal lock). Quaternions are a possible representation of attitudes—which lie in $R^{4}$—and are free of discontinuities and singularities, in addition to being more computationally efficient and numerically stable. To represent valid attitudes, they must be unit quaternions. Unit quaternions double cover the SO(3), as q and −q represent the same attitude. However, by enforcing that $q_{0}\geq 0$, we can ensure there is a one-to-one correspondence between rotation matrices and quaternions [29].

### 4.2. Self-Attention Network Design

**Figure 1:**
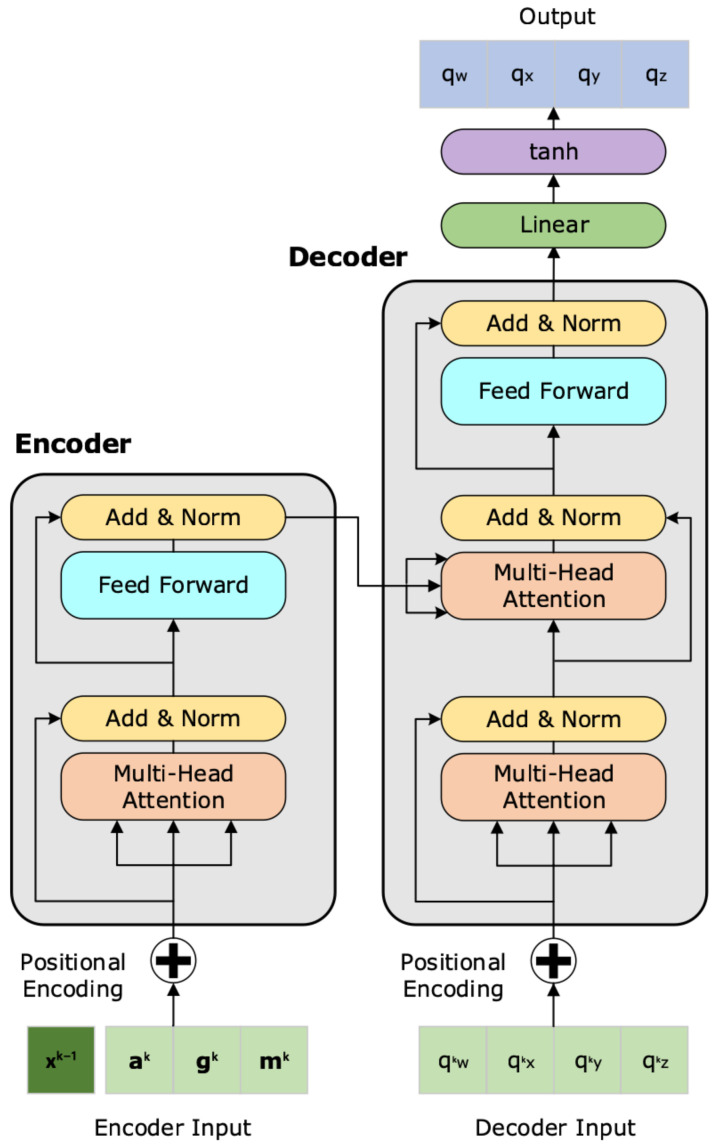
Attformer/UKF–Attformer Structure. The Attformer was trained solely with the input features of the raw three-axis measurements from the accelerometer (Equation (3)), gyroscope (Equation (1)) and magnetometer (Equation (4)). The UKF–Attformer was trained with the additional input feature of the prior UKF attitude estimate from Section 4.3.2.

Here, we formulate the Attformer and UKF–Attformer. Our models follow the original self-attention-based network, termed Transformer, proposed in [25], with an encoder–decoder structure, where both the encoder and decoder are composed of identical blocks. The filter uses a quaternion representation of attitude, allowing accelerometer and magnetometer measurements to be leveraged analytically in gradient optimisation to compute and retain information pertaining to gyroscope error and bias. To adapt the Transformer for quaternion-parameterised attitude estimation, some modifications were made. The NLP specific designs, such as the embedding and soft-max layers, are omitted, and the raw IMU measurements are used as input. The Mean Square Error (MSE) between individual quaternion components, defined in Equation (21), is applied as the loss function. The Attformer and UKF–Attformer architecture is shown in Figure 1, where the difference in each model is only in the encoder input features.

#### 4.2.1. Input and Positional Encoding

The raw information from the IMU embedded in the smartphone is used as the input state vector, I, for the Attformer:

(7) $$I\left(k\right)=\left[g_{k},\;a_{k},\;m_{k}\right]$$

and for the UKF–Attformer, with the addition of the prior UKF estimate:

(8) $$I\left(k\right)=\left[x_{k-1},\;g_{k},\;a_{k},\;m_{k}\right]$$

Unlike RNNs, self-attention-based networks are not characterised by recurrence or convolution, and as such, must utilise positional encoding in the input embeddings to model and maintain the sequential information. Giving the input vector, sequential context is necessary as the multi-head attention layer is a feed-forward layer and computes each time-step independently. Positional encoding with sine and cosine functions [30] are used to encode sequential information. This work follows [25] in using sine and cosine functions of different frequencies to embed position into the input sequences, following

(9) $$\begin{array}{cc} PE_{\left(pos,2i\right)} & =\sin\left(pos/10000^{2i/D_{\mathrm{model}\;}}\right)\\ PE_{\left(pos,2i+1\right)} & =\cos\left(pos/10000^{2i/D_{\mathrm{model}\;}}\right) \end{array}$$

where pos denotes the position, *i* the dimension and $D_{\mathrm{model}\;}$ is the model dimensionality; in this work $D_{\mathrm{model}\;}=64$.

#### 4.2.2. Encoder

The element-wise addition of the input vector and positional encoding vector is fed into two identical encoder layers. Each encoding layer is made up of two sub-layers: a multi-head attention (MHA) sub-layer and a fully connected feed-forward (FF) sub-layer. Our encoder follows the Query–Key–Value model, proposed in [25], where the scaled dot-product attention used is given by

(10) $$\mathrm{Attention}\left(Q,K,V\right)=\mathrm{softmax}\left(\frac{QK^{T}}{\sqrt{D_{k}}}\right)V$$

where queries $Q=I\left(k\right)W^{Q}\in R^{N\times D_{k}}$, keys $K=I\left(k\right)W^{K}\in R^{M\times D_{k}}$ and values $V=I\left(k\right)W^{V}\in R^{M\times D_{v}}$; each W is the respective weight matrices updated during training, and N,M denote the lengths of queries and keys (or values) and $D_{k},D_{v}$ denote the dimensions of keys (or queries) and values. The MHA consists of *H* different sets of learned projections instead of a single attention function as

$$\mathrm{MultiHeadAttn}\left(Q,K,V\right)=\mathrm{Concat}\left({\mathrm{head}}_{1},\ldots ,{\mathrm{head}}_{H}\right)W^{O}$$

where $\;{\mathrm{head}}_{i}=$ Attention $\left(QW_{i}^{Q},KW_{i}^{K},V_{i}^{V}\right)$. The projections are parameter matrices $W_{i}^{Q}\in R^{D_{\mathrm{model}\;}\times D_{k}},W_{i}^{K}\in R^{D_{\mathrm{model}\;}\times D_{k}},W_{i}^{V}\in R^{D_{\mathrm{model}\;}\times D_{v}}$ and $W^{O}\in R^{hD_{v}\times D_{\mathrm{model}\;}}$. In this work, we employ h=2 parallel attention layers, or heads. For each, we use $D_{k}=D_{v}=D_{\mathrm{model}\;}/h=32$.

In addition to the attention sub-layers, each encoder/decoder layer consists of a fully connected FF network, consisting of linear transformation and activation functions. In place of the Rectified Linear Unit (ReLU) activation function, commonly used in Transformer FF networks, we use a LeakyReLU [31] activation as follows

$$\mathrm{LeakyRe}LU\left(x\right)=\left\{\begin{array}{cc} x, & \;\mathrm{if}\;x\geq 0\\ 1\times 10^{-3}\;\cdot \;x, & \;\mathrm{otherwise}\; \end{array}\right.$$

The point-wise FF network is a fully connected module

(11) $$\mathrm{FFN}\left(H^{\prime }\right)=\mathrm{LeakyRe}LU\left(H^{\prime }W^{1}+b^{1}\right)W^{2}+b^{2}$$

where $H^{\prime }$ is the output of the previous layer, $W^{1}\in R^{D_{m}\times D_{f}}$, $W^{2}\in R^{D_{f}\times D_{m}},b^{1}\in R^{D_{f}}$ and $b^{2}\in R^{D_{m}}$ are trainable parameters, and $D_{f}$ denotes the inner-layer dimensionality. Each sub-layer has a Layer Normalisation Module inserted around each module. That is,

(12) $$H^{\prime }=\mathrm{LayerNorm}\left(\mathrm{SelfAttn}(X)+X\right)$$

where SelfAttn(·) denotes self-attention module and LayerNorm(·) the layer normal operation. The 9-dimensional (Attformer) or 13-dimensional (UKF–Attformer) resultant vector is then fed into the decoder.

#### 4.2.3. Decoder

The decoder is composed of 2 identical layers. The decoder contains the sub-layers found in the encoder, with the addition of a third sub-layer that performs multi-head attention over the output vector from the encoder. Similarly, a residual connection is employed around each sub-layer, followed by a normalisation layer. The self-attention mechanism in the decoder stacks prevents positions from influencing subsequent positions to ensure that predictions for $q_{k}$ can depend only on the known outputs at or before $q_{k-1}$. The output maps the final layer into the estimated quaternion through a hyperbolic tangent.

### 4.3. Baselines

In this section, we formulate the baselines used in this work. A GRU was built, as previous work has shown that it outperforms Temporal Convolutions Networks and other RNN variants [32]. Additionally, we use a UKF, proven effective in attitude estimation in previous work [6].

#### 4.3.1. Gated Recurrent Unit

**Figure 2:**
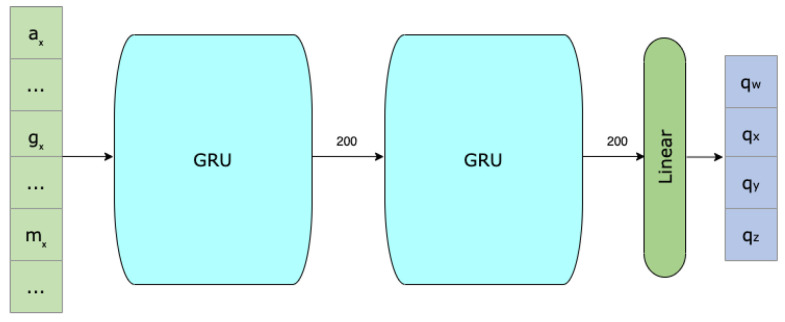
Structure of the 2-Layer GRU with 200 neurons per layer.

A stacked 2-layer GRU structure, based on [24], shown in Figure 2, which transforms the 9-dimensional IMU input, I(k), to an $N_{n}$-dimensional feature vector, where $N_{n}=200$, is the number of neurons per layer.

Note that this model differs from the GRU used in [24], as we consider magnetometer input, and the output of the network is not strictly forced to have magnitude 1. We found that using unit quaternions as the ground truth, the regressed quaternion estimate does not diverge too much from the unit norm.

#### 4.3.2. Unscented Kalman Filter

Here, we formulate the UKF based on the work in [33], where the quaternion-based UKF can be found. In this application, the dynamic model represents a physically based parametric model, and the initial attitude ( at k=0) is assumed to be known.

At time *k*, the UKF is, i=1,…,d.

(13) $$\begin{array}{cc} \chi_{0,k-1|k-1} & =x_{k-1|k-1} \end{array}$$

(14) $$\begin{array}{cc} \Delta \chi_{i,k-1|k-1} & =d^{1/2}p_{i} \end{array}$$

(15) $$\begin{array}{cc} \chi_{i,k-1|k-1} & =x_{k-1|k-1}+\Delta \chi_{i,k-1|k-1} \end{array}$$

(16) $$\begin{array}{cc} \chi_{i+d,k-1|k-1} & =x_{k-1|k-1}-\Delta \chi_{i,k-1|k-1} \end{array}$$

where $p_{i}$ is the *i*-th column of ${\left(P_{xx,k-1|k-1}+Q_{k}\right)}^{1/2}$ and $P_{xx,\;\cdot \;|\;\cdot \;}$ is the covariance matrix of $x_{\;\cdot \;|\;\cdot \;}$.

Then, the weights are

(17) $$\begin{array}{c} w_{0}=\frac{1}{d},\;w_{i}=w_{i+d}=\frac{1}{2d} \end{array}$$

The prediction step and measurement update step are given as follows:

(18) $$\begin{array}{cc} x_{k|k-1} & =\sum_{i=0}^{2d}w_{i}g\left(\chi_{i,k-1|k-1}\right)\\ P_{xx,k|k-1} & =\sum_{i=0}^{2d}w_{i}\left(\chi_{i,k|k-1}-x_{k|k-1}\right){\left(\chi_{i,k|k-1}-x_{k|k-1}\right)}^{T}\\ y_{k|k-1} & =\sum_{i=0}^{2d}w_{i}h\left(\chi_{i}^{k-1|k}\right)\\ P_{yy}^{k|k-1} & =\sum_{i=0}^{2d}w_{i}\left(h\left(\chi_{i}^{k-1|k}\right)-y_{k|k-1}\right){\left(h\left(\chi_{i}^{k-1|k}\right)-y_{k|k-1}\right)}^{T}+C_{k}\\ P_{xy}^{k|k-1} & =\sum_{i=0}^{2d}w_{i}\left(\chi_{i}^{k|k-1}-x_{k|k-1}\right){\left(h\left(\chi_{i}^{k-1|k}\right)-y_{k|k-1}\right)}^{T} \end{array}$$

where $P_{xy,\;\cdot \;|\;\cdot \;}$ is the covariance matrix of $x_{\;\cdot \;|\;\cdot \;}$ and $y_{\;\cdot \;|\;\cdot \;}$.

Finally, the correction step is

(19) $$\begin{array}{cc} S_{k} & =P_{xy}^{k|k-1}{\left(P_{yy}^{k|k-1}\right)}^{-1}\\ x_{k|k} & =x_{k|k-1}+S_{k}\left(z_{k}-y_{k|k-1}\right)\\ P_{xx}^{k|k} & =P_{xx}^{k|k-1}-S_{k}P_{yy}^{k|k-1}S_{k}^{T}. \end{array}$$

By leveraging the true attitude representations using the genetic algorithm [34], we were able to calculate optimal covariance parameters for the UKF in this work.

## 5. Dataset and Training

The dataset used in training was made publicly available by Chen et al. [35]. The dataset contains 158 sequences, totalling more than 42 km in total distance and incorporates a variety of attachments, activities and users to best reflect the broad use cases seen in real life. The data were captured via five different users and four different types of off-the-shelf consumer smartphones. The IMU data were collected and synchronised with a frequency of 100 Hz, which is generally accepted in various applications and research [36,37,38]. The ground truth in these collections was taken with a Motion Capture system. The dataset was randomly divided into training, validation and test sets, following [39]. A single sequence was left out for each of the variables as a means of unseen comparison with other techniques. The neural network is optimised and trained on the training set. After an entire epoch, the network is evaluated on the validation set as a measure of improvement. The test set provides an unbiased evaluation on the resultant network. To avoid overfitting and to improve compute efficiency, we used a sliding window to capture 100 measurements every 50 to feed into the encoder. This gave us 63,614 training samples, 18,175 validation samples and 9089 test samples. Random search algorithm was used to optimise the parameter tuning during the training process. The implementation of all adaptations was carried out with PyTorch. The training was conducted for 300 epochs, with a learning rate of 0.001, an ADAM optimiser and a dropout of 0.2. The training was conducted in parallel on 4× Nvidia V100 GPUs, made possible with the assistance of resources and services from the National Computational Infrastructure (NCI), which is supported by the Australian Government.

### 5.1. Loss Function

The loss function that is minimised during the training process in each of the models in this work is the Mean Square Error (MSE) loss function, as defined in Equation (21), where ${\hat{q}}_{i}-q_{i}$ is the element-wise subtraction of the true and estimated quaternions, respectively, and the inner product is defined as

(20) $$\langle q_{0},q_{1}\rangle =w_{0}w_{1}+x_{0}x_{1}+y_{0}y_{1}+z_{0}z_{1},$$

(21) $$\ell \left({\hat{q}}_{i},q_{i}\right)=\frac{1}{N}\sum_{i=1}^{N}l_{i},\;l_{n}=\langle {\hat{q}}_{n}-q_{n},{\hat{q}}_{n}-q_{n}\rangle$$

and *N* is the batch size.

### 5.2. Evaluation Metrics

We evaluate the above approaches using the following metrics:

(1) Inner Product (IP) of Unit Quaternion:

To give an approximate measure of dissimilarity between pairs, we need to define a distance metric. Defining the quaternion pairs as $q_{0}$ and $q_{1}$, it is possible to derive a geodesic metric for unit quaternion representation in SO(3). A simple measure used for pose estimation in [40] is defined using the angle formed by a pair of 4D unit quaternions, related to the inner product by its cosine:

(22) $$\alpha =\cos^{-1}\left(\langle q_{0},q_{1}\rangle \right)$$

where the length of the geodesic path on the 4D unit sphere is proportional to α. However, using Equation (22) results in numerical issues, as there is a discontinuous gradient in the interval (−1,1) at point 0, which results in extreme values at the points where $\cos^{-1}\left(\langle q_{0},q_{1}\rangle \right)\to 0$. We follow [41] in eliminating the inverse cosine function and define the error metric function:

(23) $$\mathrm{IP}=\frac{1}{N}\sum_{i=1}^{N}1-\left|\langle {\hat{q}}_{i},q_{i}\rangle \right|$$

for a sequence of *N* samples, where the quaternion estimate and truth at sequence *i* are given by $q_{i}$ and ${\hat{q}}_{i}$, respectively. Equation (23) computes the approximated distance metric between two unit quaternions.

(2) Root Mean Square Error (RMSE):

The RMSE metric used in this work is calculated using the following equation:

(24) $$\mathrm{RMSE}=\sqrt{\frac{1}{N}\sum_{i=1}^{N}{\left({\hat{q}}_{i}-q_{i}\right)}^{2}}$$

and *N* is the number of samples. This metric is given as the Mean Square Error (MSE) and served as our heuristic for training all of the models in this work. RMSE is widely considered a staple for evaluating the usefulness and accuracy of a model.

(3) Angular Distance Between Two Quaternions:

A quaternion can be defined by an axis in three dimensions $\left(u_{a},u_{b},u_{c}\right)$ and an angle of rotation, $\theta_{q}$, as

(25) $$q=\cos\left(\frac{\theta_{q}}{2}\right)+\sin\left(\frac{\theta_{q}}{2}\right)\left(u_{a}i+u_{b}j+u_{c}k\right)$$

Given our network estimates, each quaternion is in the form q=w+xi+yj+zk, where *w* is the real part, and the angle of q can be solved through $\theta_{q}=2\cos^{-1}\left(w\right)$. Consider again our true and estimated quaternions to be ${\hat{q}}_{i}$ and $q_{i}$, respectively, and the product $p={\hat{q}}_{i}\bar{q_{i}}$. As our estimate q approaches the truth attitude $\hat{q_{i}}$, the angle of p↦0. We can then define the angular distance between two unit quaternions as

(26) $$\theta_{p}=2\cos^{-1}p\left(R\right)$$

Given how we parameterised our network output, and to conceptually aid the reader, this is the primary metric used in our evaluation.

## 6. Evaluation

To evaluate our approach, we compare the performance of the Attformer and UKF–Attformer against a UKF, the iOS CoreMotion API and a GRU trained on the same data. We consider unseen sequences from four different users, six different activities and three different smartphones. Each method is evaluated on identical, chronologically synced data sequences, in their entirety. Each trajectory occurs over a minimum of three minutes, which allows for accumulated drift and poor solutions to significantly affect the error metrics—discussed in Section 5.2.

Table 1 demonstrates that each approach outperforms the CoreMotion’s estimates by a significant margin. The Attformer also comprehensively beats the 2-layer GRU over every metric and activity. As expected, a major issue we found with the purely end-to-end approach of the Attformer is that the network had no way of retaining the prior estimate, which led to large outlier estimates that adversely affected the performance. The combined approaches (UKF and Attformer), wherein the prior UKF was used an an input feature in the learning process, not only eliminated the outlier estimates but provided a much better estimation than either the UKF or Attformer alone.

The GRU and Attformer RMSE results in Table 1 demonstrate that both models are equally sensitive to measurement fluctuations, commonly found in smartphone inertial data. This is despite being optimised on minimising MSE. As we mentioned earlier, this is due to the large error spikes in each model’s estimate inflating these values. The separation between the GRU and Attformer is very apparent when looking at IP and angular distance, as the self-attention mechanism is able to better capture the overarching biases and drift. The Attformer estimate is consistently significantly lower over each user, device and activity for both of these metrics. GRUs, and RNNs in general, carry the inductive biases of temporal invariance and locality via their Markovian structure [42], whereas a self-attention-based design is able to minimise assumptions about the structural information of incoming data. Additionally, the attention mechanism allows for retention of the measurement noise characteristics throughout the learning process—allowing for more consistent and accurate estimates.

The RMSE of the UKF estimate is unsurprisingly lower than the Attformer and GRU models due to its probabilisitic iterative design, smoothing subsequent measurements. It also outperforms the GRU over every metric and sequence. We also observe that it outperformed the Attformer in angular distance for Users 2 and 3, which is most likely attributed to the filter covariance parameters for those particular sequences being closest to the ones calculated using the total dataset. Furthermore, we observe that large outlier noise spikes impact the Attformer far more than the UKF, evidenced by the RMSE of the UKF and Attformer compared with their respective distance and IP. This is attributed to the traditional approaches of the aforementioned prior state knowledge. By adding this knowledge to the Attformer, as the UKF–Attformer, we solidified this hypothesis, as we see dramatic improvements in every evaluation metric, particularly RMSE. Each of our performance metrics indicate that use of priors in the input feature provided a more precise and robust solution. However, paramount to the success of an attitude estimation method is not just accuracy but generalisability. We see the Attformer provides a much more generalisable solution over the GRU and UKF. The standout attitude estimation is with the UKF–Attformer, where we see that giving the NN prior estimate knowledge in the learning process eliminated the error spikes we saw in the Attformer estimates.

As a measure of generalisability for each approach, we take the mean of the angular distance over each unseen sequence, user and activity. The inbuilt iOS had a mean angular distance from the true attitude of $117.31^{\circ }$; the GRU $21.90^{\circ }$, the UKF $16.38^{\circ }$, the Attformer $16.28^{\circ }$ and, finally, the UKF–Attformer had a mean angular distance of $10.86^{\circ }$. Not only do the self-attention-based techniques outperform previous DL and parameter-optimised state-of-the-art mathematical solutions but also provide a more generalisable solution without the need for context-specific parameter tuning or prior knowledge.

## 7. Conclusions

This paper proposes a novel approach for end-to-end attitude estimation leveraging the self-attention mechanism in machine learning. We trained on a publicly available smartphone dataset, comprising triaxis accelerometer, gyroscope and magnetometer data, with Motion Capture to obtain the ground truth. We compared the performance of two self-attention approaches with a 2-layer GRU, UKF and the iOS CoreMotion API. Each approach was evaluated over a range of unseen sequences from different users, devices and activities. We showed that the self-attention method outperforms previous data-driven techniques that rely on RNNs, as they are unable to capture the long-term dependencies in the data. We showed that the self-attention mechanism’s well-known ability to retain information and dependencies over long sequences improved our attitude estimation solution. Additionally we showed that providing the network with prior state knowledge, through the use of a UKF, dramatically improves the network’s estimate. Both self-attention methodologies with and without prior state information proposed in this work provide a stable, accurate and generalisable solution, with an average angular distance from truth of $10.86^{\circ }$ and $16.28^{\circ }$, respectively. The UKF, GRU and iOS averages were $16.38^{\circ }$, $21.90^{\circ }$ and $117.31^{\circ }$. Future work will focus on the limitations of the algorithm and involve further developing the framework into an end-to-end inertial odometry solution. 

## Figures and Tables

**Table 1:** Attitude error metric comparison over each full, unseen activity sequence. The best performing approach over each sequence and for each metric has been made bold to aid the reader.

	User 2			User 3			User 4		
Model	RMSE	Distance (∘)	IP	RMSE	Distance (∘)	IP	RMSE	Distance (∘)	IP
iOS	0.599	129.34	0.612	0.640	130.20	0.482	0.696	127.99	0.580
GRU	0.294	19.98	0.163	0.241	17.56	0.114	0.285	20.39	0.148
UKF	**0.109**	7.34	0.024	0.160	7.84	0.052	0.259	17.84	0.135
Attformer	0.297	13.20	0.010	0.250	13.30	0.024	0.305	11.28	**0.017**
UKF-Att	0.124	**7.31**	**0.005**	**0.138**	**7.13**	**0.000**	**0.184**	**9.61**	0.020
	**User 5**			**Pocket**			**Running**		
	**RMSE**	**Distance (∘)**	IP	**RMSE**	**Distance (∘)**	IP	**RMSE**	**Distance (∘)**	IP
iOS	0.735	129.60	0.607	0.677	128.03	0.720	0.657	91.86	0.533
GRU	0.265	20.12	0.162	0.195	13.76	0.069	0.144	14.04	0.030
UKF	0.145	13.58	0.042	0.145	10.97	0.042	0.150	15.19	0.065
Attformer	0.267	9.04	0.030	0.120	7.71	0.042	0.144	11.29	0.022
UKF-Att	**0.135**	**8.99**	**0.008**	**0.085**	**7.06**	**0.005**	**0.114**	**8.46**	**0.012**
	**Slow Walking**			**Trolley**			**Handbag**		
	**RMSE**	**Distance (∘)**	IP	**RMSE**	**Distance (∘)**	IP	**RMSE**	**Distance (∘)**	IP
iOS	0.612	90.60	0.667	0.600	128.99	0.585	0.606	93.81	0.629
GRU	0.136	10.34	0.030	0.298	21.44	0.188	0.155	16.25	0.038
UKF	0.123	14.83	0.048	0.205	10.31	0.084	0.147	17.80	0.058
Attformer	0.171	8.13	0.020	0.359	12.83	**0.001**	0.145	10.99	0.017
UKF-Att	**0.112**	**6.64**	**0.014**	**0.160**	**7.91**	0.020	**0.098**	**10.31**	**0.011**
	**Handheld**			**iPhone 5**			**iPhone 6**		
	**RMSE**	**Distance (∘)**	IP	**RMSE**	**Distance (∘)**	IP	**RMSE**	**Distance (∘)**	IP
iOS	0.596	130.09	0.627	0.602	128.72	0.591	0.602	129.14	0.559
GRU	0.450	75.99	0.421	0.348	24.06	0.193	0.337	23.81	0.204
UKF	0.454	61.90	0.415	0.181	**9.51**	0.067	0.264	**9.47**	0.140
Attformer	0.483	65.25	0.234	0.411	20.84	0.035	0.384	21.27	0.036
UKF-Att	**0.341**	**35.09**	**0.100**	**0.169**	10.27	**0.020**	**0.249**	11.55	**0.030**

## Data Availability

The publicly available dataset analysed in this work can be found here: [35].
